# Custom orthotic design by integrating 3D scanning and subject-specific FE modelling workflow

**DOI:** 10.1007/s11517-024-03067-2

**Published:** 2024-03-06

**Authors:** Yinghu Peng, Yan Wang, Qida Zhang, Shane Fei Chen, Ming Zhang, Guanglin Li

**Affiliations:** 1 CAS Key Laboratory of Human-Machine Intelligence-Synergy Systems, Shenzhen Institutes of Advanced Technology, Chinese Academy of Sciences, Shenzhen, China; 2 Department of Biomedical Engineering, Faculty of Engineering, Hong Kong Polytechnic University, Hong Kong, 999077 SAR China; 3 Hong Kong Polytechnic University Shenzhen Research Institute, Shenzhen, 518057 China; 4 Research Institute for Sports Science and Technology, The Hong Kong Polytechnic University, Hong Kong, SAR China; 5 Musculoskeletal Research Laboratory, Department of Orthopaedics & Traumatology, The Chinese University of Hong Kong, Hong Kong, SAR China

**Keywords:** Foot–ankle joint, Finite element analysis, Foot orthosis, Customized foot scaling

## Abstract

The finite element (FE) foot model can help estimate pathomechanics and improve the customized foot orthoses design. However, the procedure of developing FE models can be time-consuming and costly. This study aimed to develop a subject-specific scaled foot modelling workflow for the foot orthoses design based on the scanned foot surface data. Six participants (twelve feet) were collected for the foot finite element modelling. The subject-specific surface-based finite element model (SFEM) was established by incorporating the scanned foot surface and scaled foot bone geometries. The geometric deviations between the scaled and the scanned foot surfaces were calculated. The SFEM model was adopted to predict barefoot and foot-orthosis interface pressures. The averaged distances between the scaled and scanned foot surfaces were 0.23 ± 0.09 mm. There was no significant difference for the hallux, medial forefoot, middle forefoot, midfoot, medial hindfoot, and lateral hindfoot, except for the lateral forefoot region (*p* = 0.045). The SFEM model evaluated slightly higher foot-orthoses interface pressure values than measured, with a maximum deviation of 7.1%. These results indicated that the SFEM technique could predict the barefoot and foot-orthoses interface pressure, which has the potential to expedite the process of orthotic design and optimization.

**Graphical abstract:**

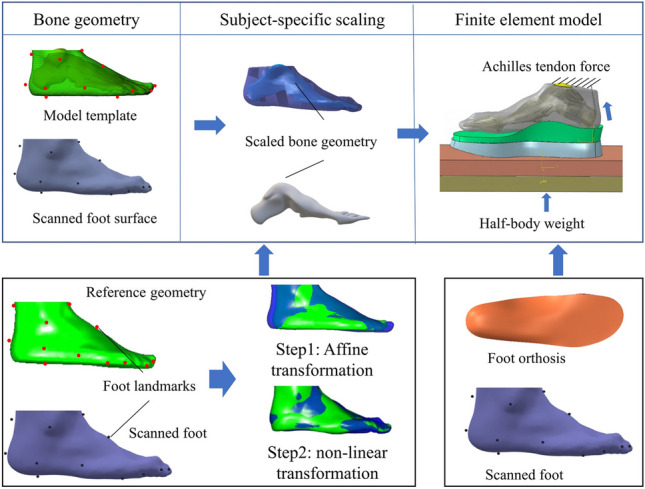

## Introduction

The prevalence of foot pain ranged from 17 to 42% among the adult population, which has been recognized as a risk factor for impaired locomotor, decreased balance, increased fall risks, and worse quality of life [1]. Foot pain could also affect foot alignment, joint kinematics, and muscle activations [2]. Due to the coupled mechanism of the lower limb joints, the dynamic instability and misalignment of the foot may lead to kinematics and kinetics alterations in the proximal joints and accelerate the onset or progression of lower limb dysfunction and pain [3, 4]. Researchers have endeavored to develop biomechanical indicators to identify the causes and severity level of foot pain, thus enabling clinicians to prevent potential damage [5–7]. Some studies have revealed that regional foot pain, such as metatarsalgia, diabetic foot ulcers, and heel pain, was associated with excessive loading in the soft tissue [8]. To reduce the potential damage of high loading on the soft tissues, researchers have sought for conservative treatments to reduce regional foot pain by redistributing foot pressure [9].

Prefabricated and customized foot orthoses have been widely adopted to manage regional foot pain for individuals with foot problems, such as hindfoot pain and diabetic foot [10, 11]. Compared to prefabricated foot orthoses, customized foot orthoses can provide better functional outcomes in pressure distribution and pain relief by considering individual differences [12, 13]. Traditionally, customized insoles were fabricated using molding and machining, which required complex manufacturing procedures, long production time, and expensive production costs [14]. Researchers attempted to manufacture customized foot orthoses with computer-aided orthosis design and 3D-printed techniques [14]. Although the procedure for producing 3D-printed foot orthoses was simpler and less expensive, it was still challenging to modify the shapes of the orthoses [14]. In that case, anticipating the functional outcomes of foot orthoses might help to avoid improperly designed orthoses. Nevertheless, customized foot orthoses were often designed and fabricated based on the foot surface geometries and the orthotist's expertise, making it difficult to predict their mechanical performance in real-world conditions [11]. The foot plantar pressure, as an essential biomechanical parameter, could be adopted to improve our understanding of foot orthoses-induced biomechanical alterations, which facilitates the development of knowledge-based treatment protocols for foot pain treatment.

Plantar pressure analysis, also known as polarography, has been used to examine the biomechanical responses of the foot to external loading circumstances [7]. To date, many polarograph devices have been developed to measure foot pressures, including barefoot and in-shoe pressure distribution [15]. The in-shoe devices were widely employed to evaluate the foot kinetics and stability under various circumstances due to their portability [16]. High-resolution in-shoe systems, such as the Pedar and F-scan systems, are often used in the evaluation of personalized foot orthoses design [17]. However, these devices are either expensive, readily slippery, or thick. Meanwhile, foot-orthoses interface pressure could not be obtained until the customized foot orthoses were made, which was not realistic in the clinical setting.

FE analysis offers an alternative to experimental measurements and has been adopted to investigate the biomechanical effects of surgical treatment strategies, implant designs, and orthosis parameters [18]. Regarding the foot orthoses designs, previous studies have proposed different foot models [19, 20]. Some studies adopted sophisticated models by incorporating the detailed foot bones, ligaments, and muscles, which could reveal the internal soft tissues’ stress/strain and foot pressures under various loading conditions [21, 22]. In contrast, the simplified foot model with merged foot bones was also proposed and could obtain reasonable foot-orthoses interface pressure [23, 24]. Although many studies have investigated the biomechanical effects of orthoses design, foot-orthoses models were still scarce in clinical settings. This could be attributed to costly and time-consuming procedures, including MRI or CT data collection, and anatomical geometries extraction [25]. In terms of personalized foot orthoses or shoe design, faster foot modelling techniques for foot-orthoses interface pressure prediction could provide important guidelines for orthosis optimization [23, 25].

This study aims to firstly propose and validate a scanned foot surface-based finite element model (SFEM) with four different customized scaling methods; and secondly, to evaluate its functional outcomes on foot orthoses design. The foot surfaces and foot pressures were collected for the model evaluations. Meanwhile, the proposed SFEM method was used for foot-orthoses interface pressure evaluation.

## Materials and methods

### Participant information

In this study, six participants (including four males and two females) were recruited for the customized foot finite element modelling (twelve feet). The inclusion criteria were the participants be (1) 18 to 30 years old, and (2) not overweighted (BMI < 30 kg·m^−2^). The exclusion criteria included severe foot abnormalities, trauma, and soft tissue damage. Before the experiment, ethical approval was approved by the Human Subjects Ethics Sub-Committee of the Hong Kong Polytechnic University (Number: HSEARS20190124008). Meanwhile, the participants were required to sign the consent form and information form for data collection.

### Experimental protocol

The geometry of the foot was collected using a 3D foot surface scanner (UPOD-HDS; ScanPod3D, Wuhan, China). The foot surfaces of both feet were scanned for each participant under minimal weight-bearing conditions. During the minimal weight-bearing scanning process, the participant sat on a chair, with the shank perpendicular to the surface of the scanner. The geometry of the scanned foot surface was processed by trimming the top (65 mm above) for the twelve feet. Meanwhile, the foot pressure measurement system F-scan (Tekscan Inc., Boston, USA) was used to obtain the foot plantar pressure of each participant. The foot pressures of both feet under balanced standing conditions were collected. The experimental setup for foot pressure collection has been reported in our previous study [26].

### Customized foot scaling

**Fig. 1:**
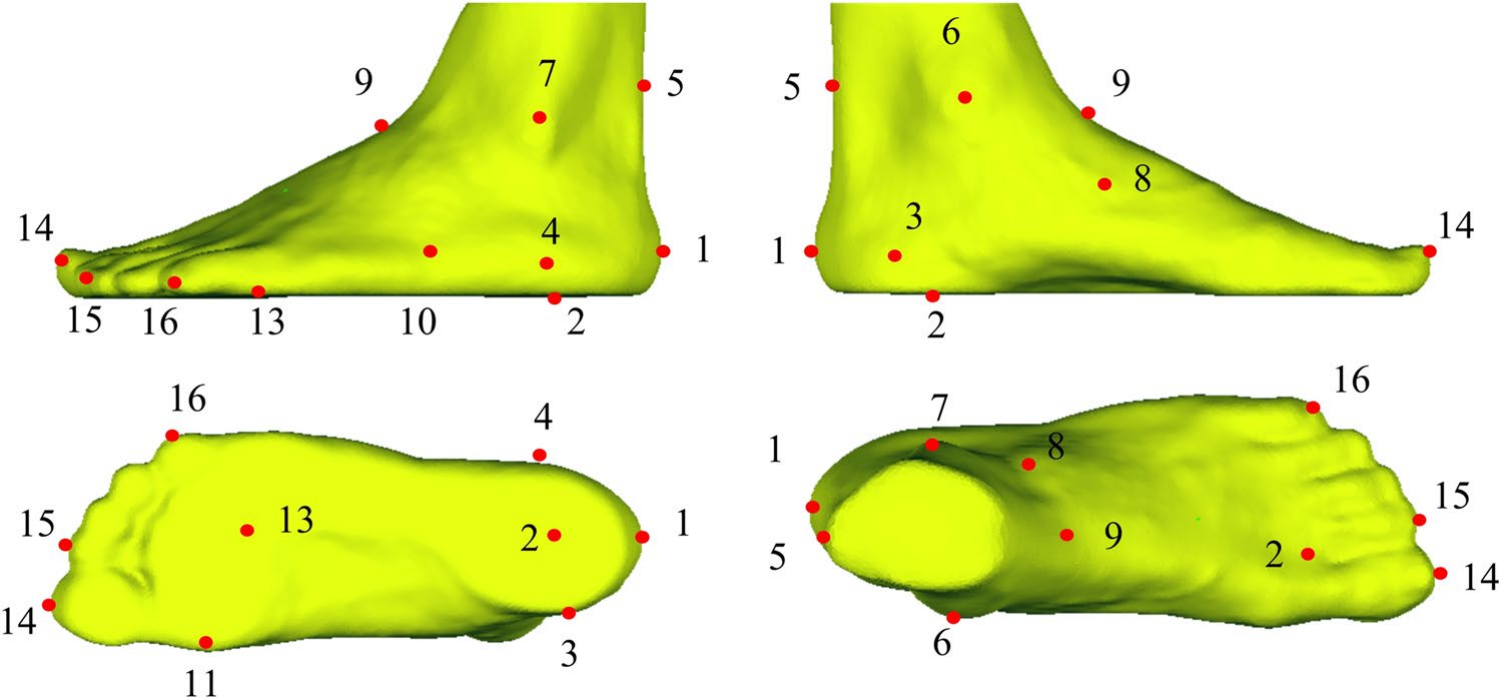
Anatomical landmark locations

**Fig. 2:**
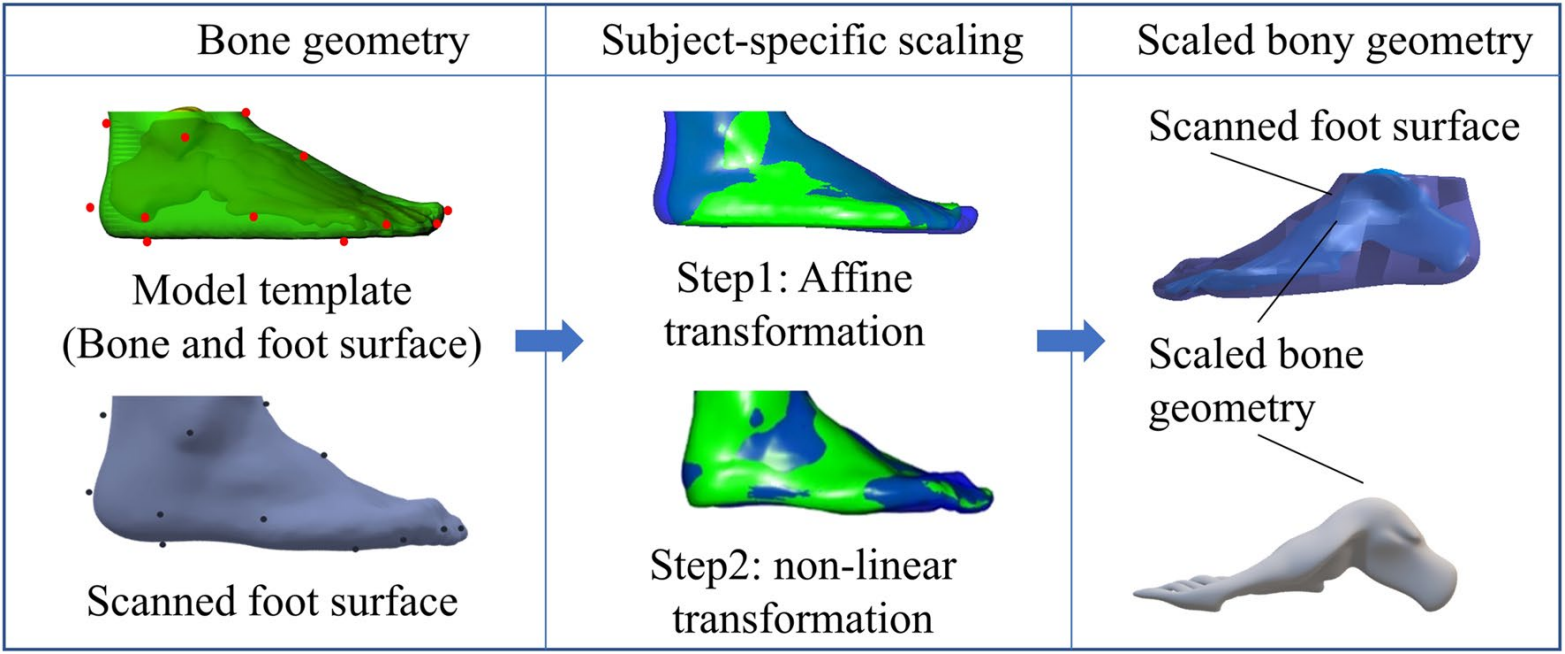
Workflow of the customized foot bone reconstrcution based on surface-based scaling method

For each participant, the collected foot surfaces were regarded as the targeted surfaces. The foot surface and bony geometries of the Glasgow-Maastricht foot model in the Anybody software (AnyBody Technology, Aalborg, Denmark, version 6.0.5) were used as the template model [27]. We merged the bony segments in the template foot model to reduce the complexity. Before the customized foot scaling, the landmarks on the template and targeted foot surfaces were selected [28]. Sixteen landmarks were determined, including points in posterior calcaneus, central plantar heel, heel medial, heel lateral, Achilles tendon between malleoli, malleolus medial, malleolus lateral, navicular tuberosity, navicular dorsal, the fifth metatarsal basis, the first metatarsal head, the second tarsometatarsal dorsal, the third metatarsophalangeal plantar, hallux tip, the second toe tip, and the fifth toe tip (Fig. 1). A non-linear scaling method was performed to obtain the scaled foot bony geometries based on the corresponding landmarks between the template and scanned foot surfaces [29]. The scaling process can be divided into several steps, including affine transformation and non-linear transformations (Fig. 2). The affine transformation was determined by the corresponding landmarks in the template and targeted foot surfaces. The function of affine transformation was defined as:

1 $$\left[ {\begin{array}{*{20}c} {P^{\prime}} \\ 1 \\ \end{array} } \right] = M\left[ {\begin{array}{*{20}c} P \\ 1 \\ \end{array} } \right]$$

Where $M$ is the $4 \times 4$ affine transformation matrix, including scaling, rotation, and translation, $P^{\prime}$ are the points coordinates on the targeted surface, $P$ are the points coordinates on the template surface.

**Fig. 3:**
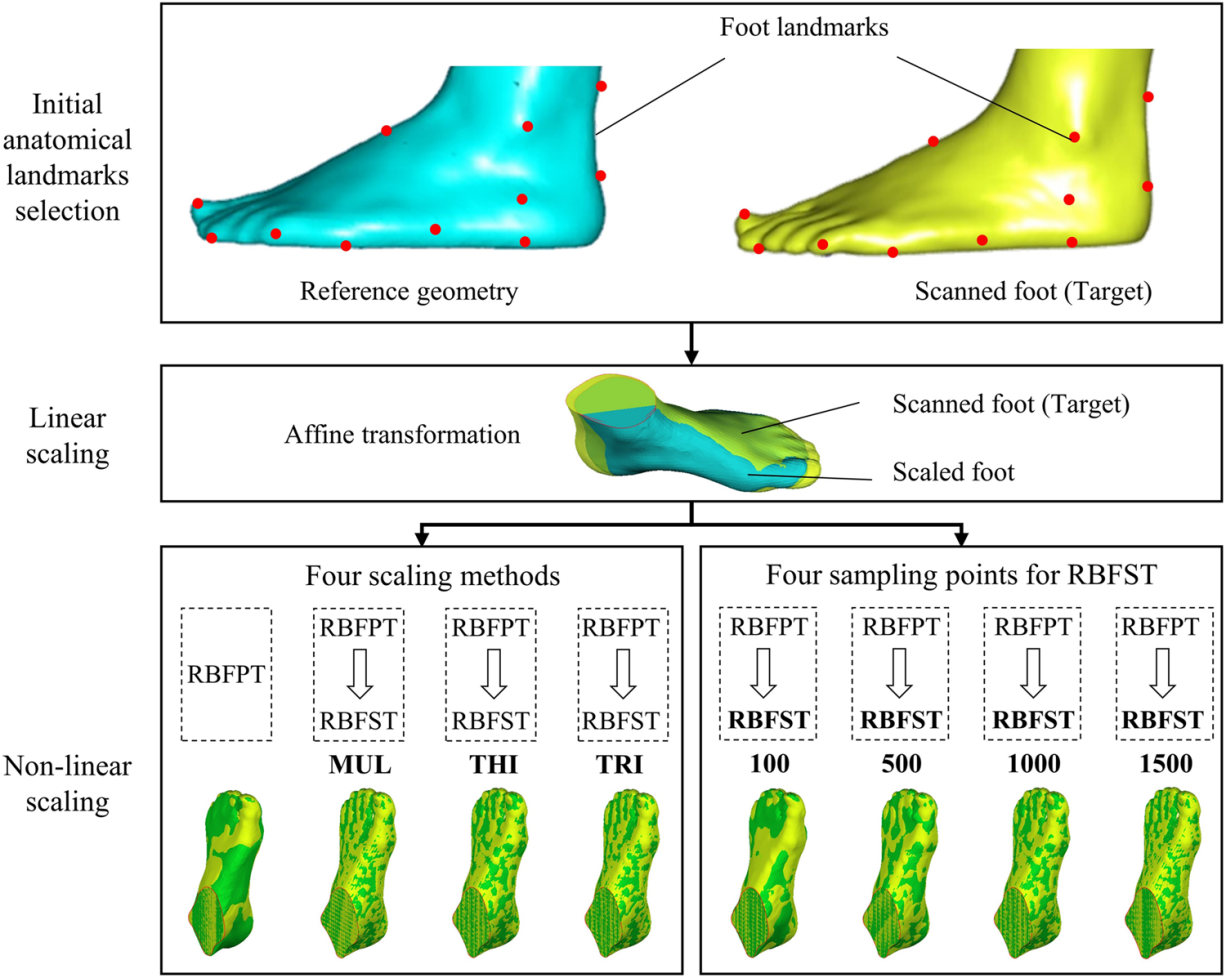
Foot customized scaling with four different scaling methods and four sampling points for RBFST. The four non-linear scaling methods including 1) RBFPT, 2) RBFPT and RBFST with thin-plate (THI) basis function, 3) RBFPT and RBFST with multi-quadratic (MUL) basis function, and 4) RBFPT and RBFST with triharmonic (TRI) basis function. The sensitivity of the seeded numbers, including 100,500,1000 and 1500 points, was investigated for the RBFST. RBFPT represents radial basis function point-based transformation; RBFST represents radial basis function surface-based transformation

The non-linear transformation methods including the built-in radial basis function (RBF) point-based transformation (RBFPT) and built-in RBF surface-based transformation (RBFST).The RBFPT was performed based on the manually selected landmarks on the template and targeted surfaces [29]. The RBFST allows topologically equivalent surfaces to be morphed using vertex-vertex correspondence, which was performed after the RBFPT step. The built-in RBFST could automatically control the density of the sampling points. In this study, the effects of four scaling procedures were analyzed, including 1) RBFPT, 2) RBFPT and RBFST with thin-plate (THI) basis function, 3) RBFPT and RBFST with multi-quadratic (MUL) basis function, and 4) RBFPT and RBFST with triharmonic (TRI) basis function (Fig. 3).

The RBFPT and RBFST adopted the non-linear scaling function to create the morphing function between the template and targeted points [29]. The RBF interpolation function for the non-linear scaling was defined as:

2 $$f^{i} (x) = \sum\limits_{j = 1}^{n} {c^{i}_{j} \Phi \left( {\left\| {x - x_{j} } \right\|} \right)}$$

where $i$ = 1,2,3 are the three-coordinate axis, $x$ is the free point for which the interpolation is evaluated, $x_{j}$ is the jth source points corresponding known target points $[f^{1} (x_{j} ) \, f^{2} (x_{j} ) \, f^{3} (x_{j} )]$, $c_{j}$ are the coefficients of the RBF functions $\Phi$, computed based on the source and target landmarks.

Three types of RBF functions $\Phi$ were adopted, including MUL basis function, THI basis function, and TRI basis function. More details about these functions can be seen in (3)-(5) [30].

MUL basis function was defined as:

3 $$\Phi (r) = \sqrt {r^{2} - \beta }$$

where $r$ is the distance between points of scaled and targeted surfaces, equal to $\left\| {x - x_{j} } \right\|$, $\beta$ is the shape parameter with default value 0.2 in the Anybody software*.*

THI basis function was defined as:

4 $$\Phi (r) = r^{2} \log (r)$$

TRI basis function was defined as:

5 $$\Phi (r) = r^{3}$$

Meanwhile, the RBFST method requires automatically selecting the corresponding points on the scaled surfaces and targets at random, which enables the morphing of topologically equivalent surfaces using vertex-vertex correspondence [30]. When the optimal non-linear transformation method was selected, the sensitivity of the selected point numbers, including 100,500,1000 and 1500 points, was investigated for the RBFST (Fig. 3). The geometric error maps between the scaled surfaces and targets were examined for the different point numbers.

### Finite element model

The foot–ankle joint geometries, including the encapsulated bulk tissue and bony geometries, were constructed in the foot models using finite element software Abaqus 6.14 (Simulia, Dassault Systemes, Johnston, RI,USA). A 2-mm-thick membrane covering the bulk soft tissue was used to represent the skin layer. The contact property between the foot surface and the ground plate was defined with a friction coefficient of 0.6 [31].

The meshes and materials were assigned to the foot–ankle joint components. The bony structure was assumed homogenous and linearly elastic with Young's modulus of 10 GPa and Poisson's ratio of 0.34 [32]. The hyperelastic material model was assigned to the encapsulated soft tissue with the second-order polynomial strain energy potential equation [33].

6 $$U = \sum\limits_{i + j = 1}^{2} {C_{ij} (\overline{{I_{1} }} - 3)^{i} (\overline{{I_{2} }} - 3)^{j} } + \sum\limits_{i = 1}^{2} {\frac{1}{{D_{i} }}(J_{e1} - 1)^{2i} }$$

where $U$ is the strain energy per unit of the reference volume; $C_{ij}$ and $D_{i}$ are the materials coefficients with $C_{10}$ = 0.8556 Nmm^−2^, $C_{01}$ = -0.0584 Nmm^−2^, $C_{20}$ = 0.03900 Nmm^−2^, $C_{11}$ = -0.02319 Nmm^−2^, $C_{02}$ = 0.00851 Nmm^−2^, $D_{1}$ = 3.65273 mm^−2^N^−1^, $D_{2}$ = 0.000 mm^−2^N^−1^; $\overline{{I_{1} }}$ and $\overline{{I_{2} }}$ are the first and second first and second deviatoric strain invariants; $J_{e1}$ is elastic volume ratio.

The $\overline{{I_{1} }}$ and $\overline{{I_{2} }}$ were defined with the form:

7 $$\overline{{I_{1} }} = \overline{\lambda }_{1}^{2} + \overline{\lambda }_{2}^{2} + \overline{\lambda }_{3}^{2}$$

8 $$\overline{{I_{2} }} = \overline{\lambda }_{1}^{ - 2} + \overline{\lambda }_{2}^{ - 2} + \overline{\lambda }_{3}^{ - 2}$$

where $\overline{\lambda }_{i}^{2}$ defines the deviatoric stretches with $J_{e1}^{ - 1/3} \lambda_{i}$, $\lambda_{1}$,$\lambda_{2}$, and $\lambda_{3}$ are the deviatoric principal stretches.

The first-order Ogen model was adopted to represent the hyperplastic material properties of skin [34].

9 $$U = \frac{2\mu }{{\alpha^{2} }}(\lambda_{1}^{\alpha } + \lambda_{2}^{\alpha } + \lambda_{3}^{\alpha } - 3)$$

where $U$ is the strain energy per unit of the reference volume; $\lambda_{1}$, $\lambda_{2}$, and $\lambda_{3}$ are the deviatoric principal stretches; $\mu$ and $\alpha$ are the material coefficients with $\mu$ = 0.122 MPa, and $\alpha$ = 18.

**Fig. 4:**
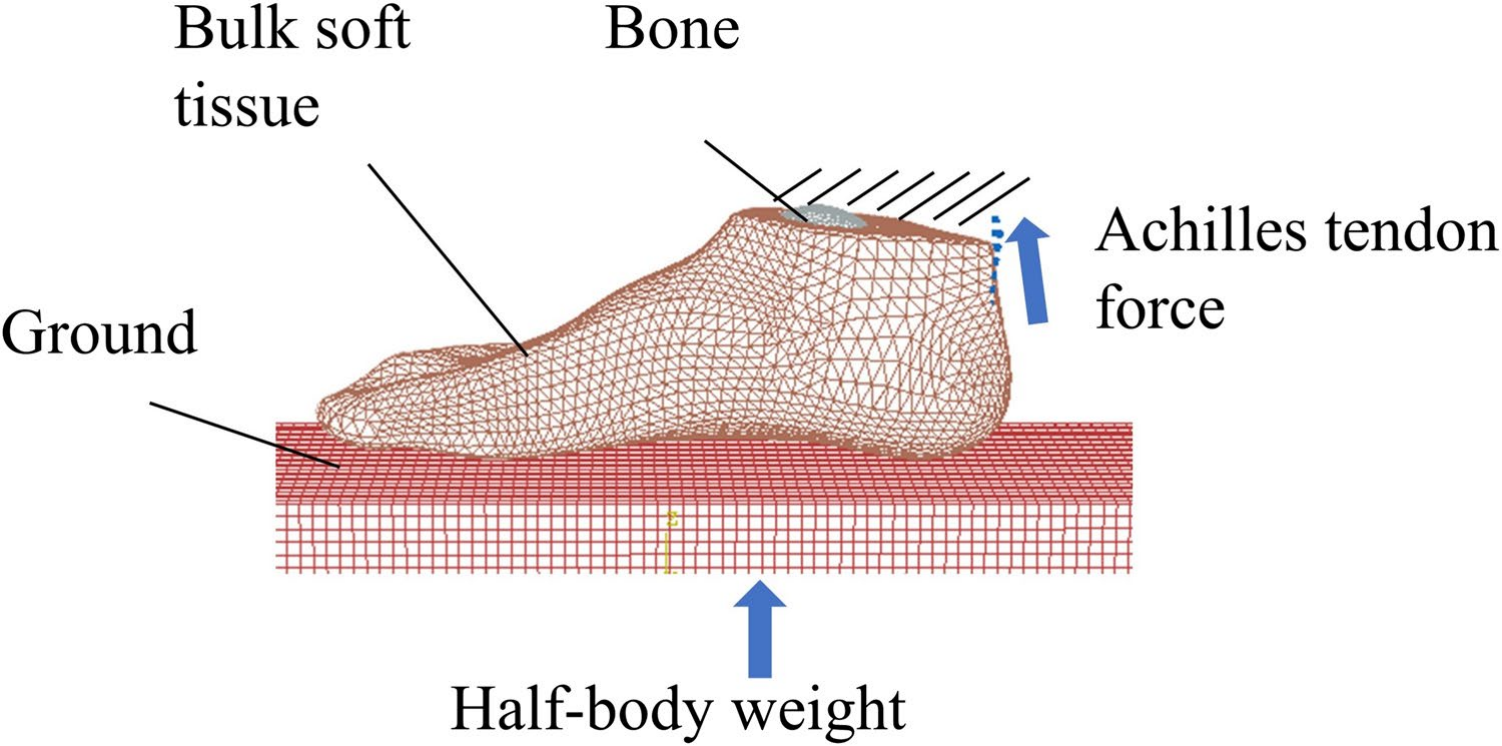
Foot finite element modelling method based on scanned foot surface

Linear hexahedra element (C3D8) and three-node triangular membrane element (M3D3) were assigned to the ground plate and skin layer, respectively. Linear tetrahedral element (C3D4) was assigned to the bones and the encapsulated bulk tissue. The simulation model exerted a vertical force vector on the ground plate, which was equivalent to half of the body weight. A quarter of the body weight was applied to the triceps surae muscle force during balanced standing, which was regulated by the Achilles tendon unit. The ground plate was fixed in all directions except for the vertical direction. The model constrained the proximal cross-sectional surfaces of the tibia, fibula, and skin, thereby preventing any movement in any direction. More detailed information about the SFEM can be seen in Fig. 4. The mesh convergence test was conducted in the foot model by iteratively decreasing the element size by half (from 20 to 1.25 mm). The deviations in the peak plantar pressure ranged from 1.06% to 2.0% (from 5 mm to 1.25 mm), which was regarded as acceptable with assumed criteria of less than 5% deviation [35]. The overall element size was 3 mm for the bone structures, and 5 mm for the encapsulated soft tissue and ground plate. The elements were refined locally to accommodate small-part geometries, contact regions, and abrupt geometric changes.

### Validation

The simulation of twelve feet models was conducted using the standard static solver. The foot plantar pressure distribution was predicted for each foot. The proposed SFEM models were validated by comparing the predicted foot pressures with the measurements. The foot regions were divided into seven parts, including medial heel, lateral heel, midfoot, the first metatarsal (medial forefoot), the second and third metatarsal (middle forefoot), the fourth and fifth metatarsals (lateral forefoot), and hallux. The maximum contact pressure in each region was extracted for analysis. The normality of the data was checked by the Shapiro–Wilk test. If the data was normally distributed, paired-t test will be adopted to compared the experimental and predicted values. Otherwise, Wilcoxon matched-pairs signed rank test was performed. The *p* value was set as 0.05.

### Application in foot orthoses design

**Fig. 5:**
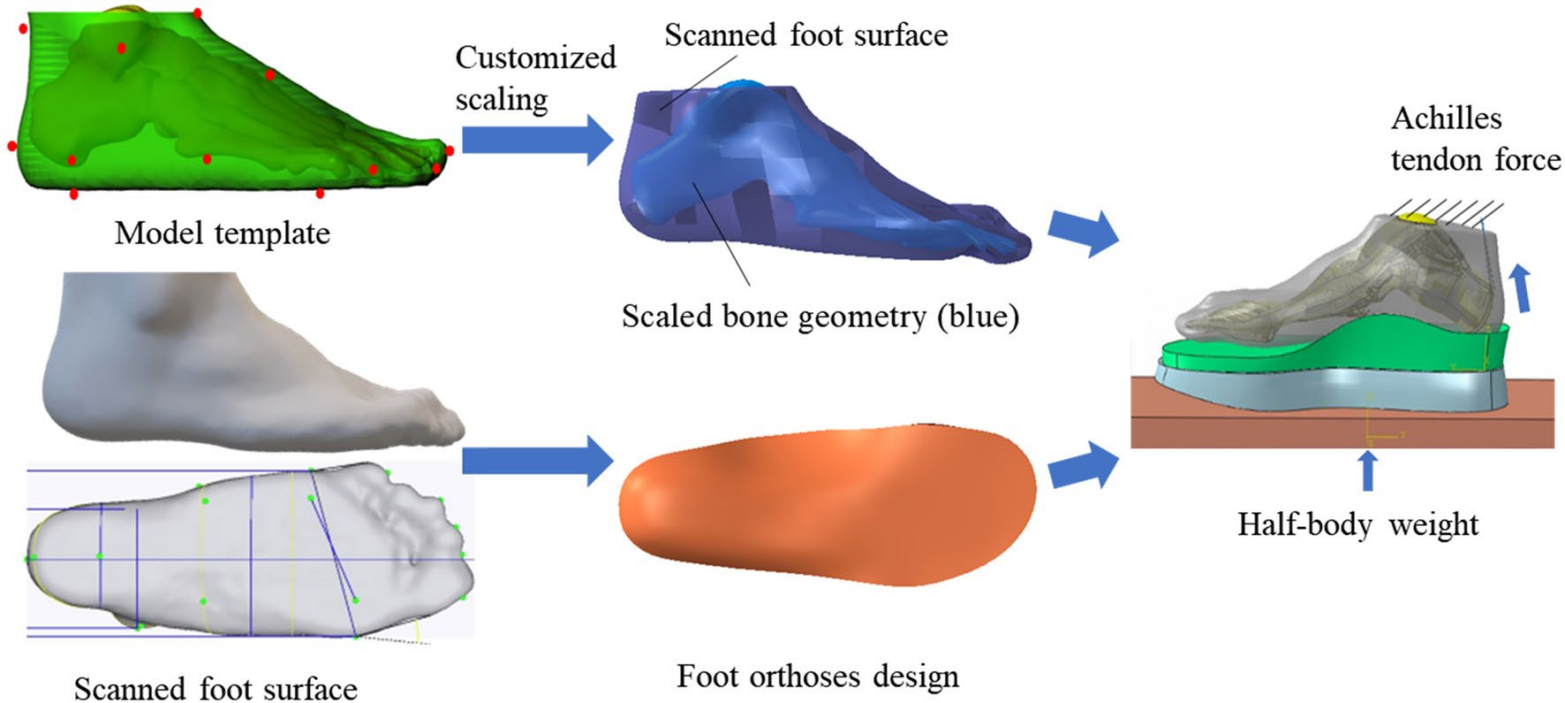
Workflow of the customized finite element modelling with surface-based scaling method for foot orthoses evaluation

For the demo application, the established SFEM model procedure was adopted for the application of foot orthosis. The SFEM approach was customized for one participant, utilizing the customized foot orthosis from our previous study [36]. Firstly, the foot surface and scaled bony geometry were obtained to establish the foot finite element model based on the proposed non-linear morphing method from Section 2.3. Secondly, the foot orthosis was designed based on the foot surface under the minimal weight-bearing condition and fabricated with 3D printed method. Finally, the foot-orthosis finite element model was established (Fig. 5). In our previous study, the foot surface, foot interface pressure, and customized 3D printed foot orthosis of one participant was collected [36]. The SFEM-based methods adopted the same foot surface, foot orthosis, boundary conditions, and material properties, and mesh types, except the scaled foot bone compared to the model in our previous studies [26, 36]. Figure 5 illustrates the procedure of foot-orthosis finite element modeling.

Based on the developed SFEM model, the foot-orthoses interface pressure was predicted. The foot-orthoses interface pressure of SFEM was compared to measurement for the recruited participant [36]*.* The maximum contact pressures in eight foot regions (medial heel, lateral heel, medial midfoot, lateral midfoot, medial forefoot, middle forefoot, lateral forefoot, and hallux) were extracted for analysis. Pearson correlation analysis and Bland–Altman plots were performed to evaluate the association and agreement, respectively, between measurements and predictions.

## Results

### The sensitivity analysis of the scaling methods

**Fig. 6:**
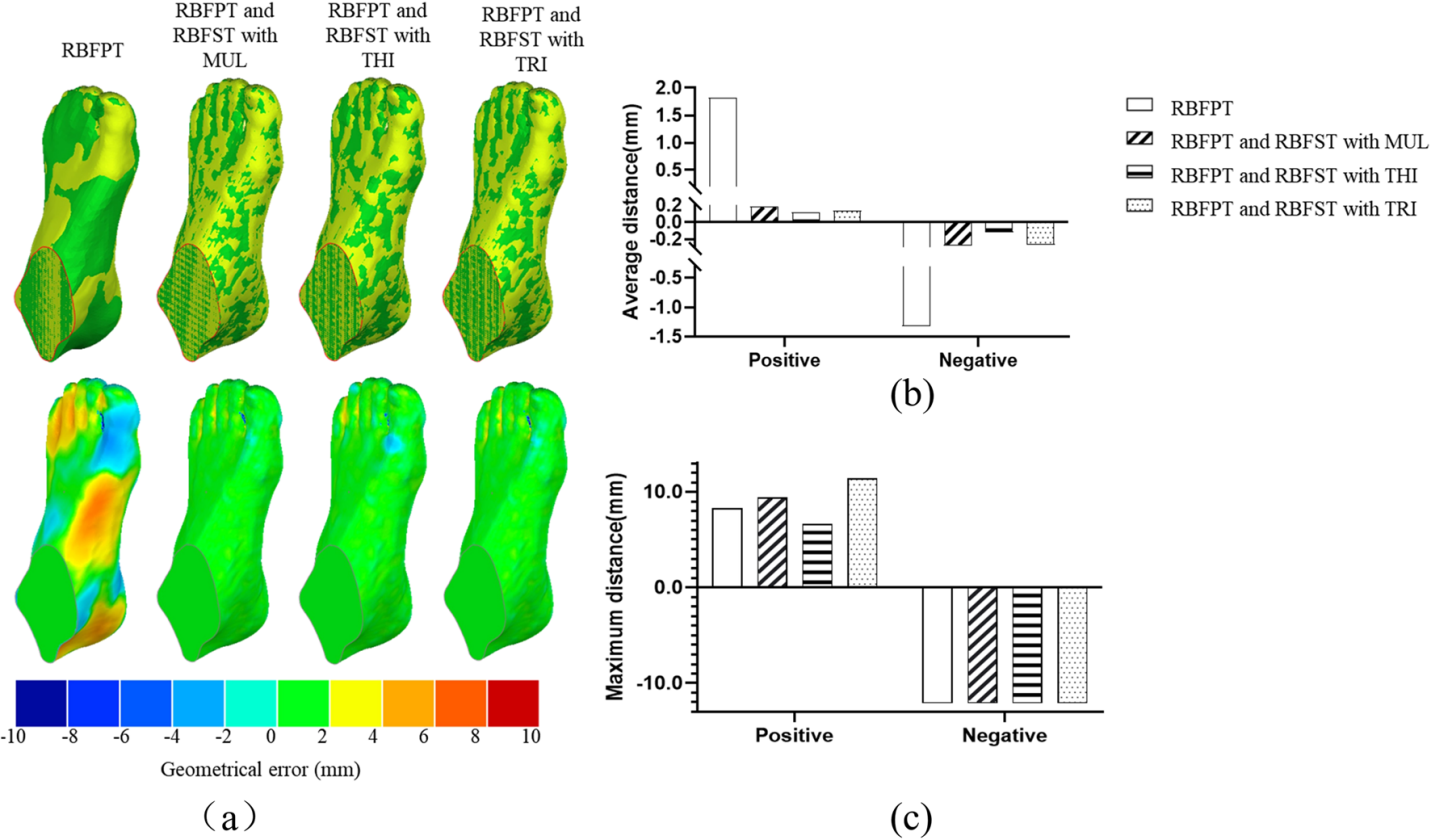
Geometric error maps between scaled and targeted foot surfaces for the four different scaling methods, including (**a**) geometric error maps, (**b**) geometric errors (averaged distance), and (**c**) geometric errors (maximum distance). RBFPT represents radial basis function point-based transformation; RBFST represents radial basis function surface-based transformation. MUL, THI, and TRI represent multi-quadratic basis function, thin-plate basis function, and triharmonic basis function, respectively. For the geometric error maps, the top indicates the scaled and targeted foot surfaces, yellow for the targeted foot surface and green for the scaled surface

The geometric error maps between scanned and scaled foot surfaces for the four different scaling methods are shown in Fig. 6(a). The yellow surface represented the targeted surface, while the green surface denoted the scaled surface. The targeted foot surface was adopted as a reference. Negative values indicated that the scaled foot surface was contained within the reference, whereas positive values indicated outer. The averaged geometric errors between the scanned and scaled foot surfaces for the four different scaling methods are illustrated in Fig. 6(b). The RBFPT and RBFST with the THI basis function achieved the best result with 0.13 mm and -0.12 mm for the positive and negative errors, respectively. The maximum geometric errors between the scanned and scaled foot surfaces for the four different scaling methods are illustrated in Fig. 6(c). The maximum negative geometric errors among these methods were similar. However, the RBFPT and RBFST with the THI basis function achieved the best result with 6.7 mm for the positive value.

**Fig. 7:**
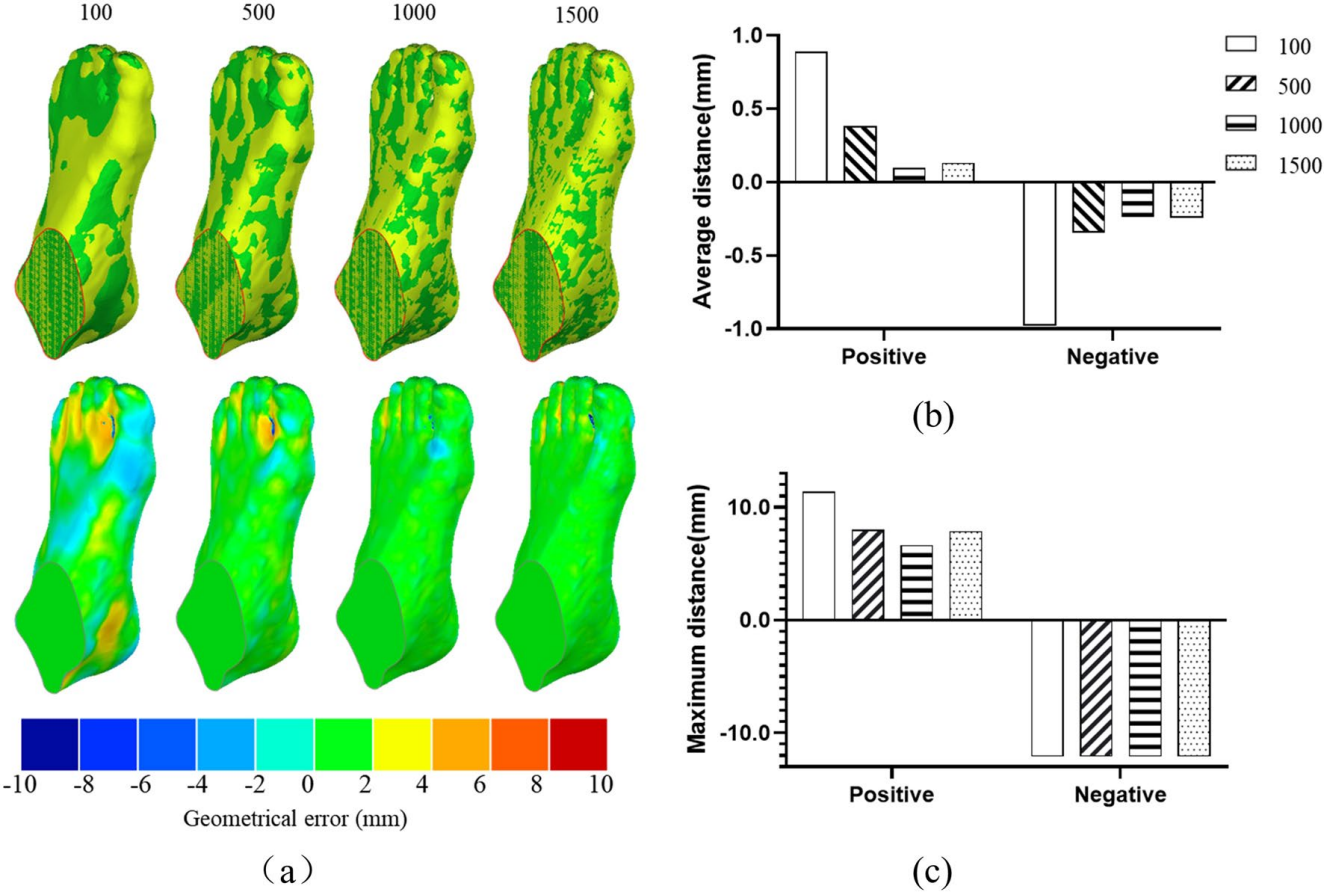
Geometric errors between scaled and targeted foot surfaces among the four different sampling points methods of RBFST, including (**a**) geometric error maps, (**b**) geometric errors (averaged distance), and geometric errors (maximum distance). For the geometric error maps, the top indicates the scaled and targeted foot surfaces, yellow for the targeted foot surface, and green for the scaled surface

The typical geometric error maps between scanned and scaled foot surfaces for four different sampling points are displayed in Fig. 7(a). The scaling method adopted was RBFPT and RBFST with the THI basis function. The averaged geometric errors between the scanned and scaled foot surface for four different sampling points are illustrated in Fig. 7(b). The geometric error was the lowest when the sampling point number is 1000.The maximum geometric errors between the scanned and scaled foot surfaces for the four different sampling points are illustrated in Fig. 7(c). The maximum negative geometric errors among these sampling points were similar. For positive results, the geometric error decreased with an increase in sampling points from 100 to 1000. However, the geometrical error increased, especially in the plantar side of toes areas, when with the sampling points increased from 1000 to 1500.

**Fig. 8:**
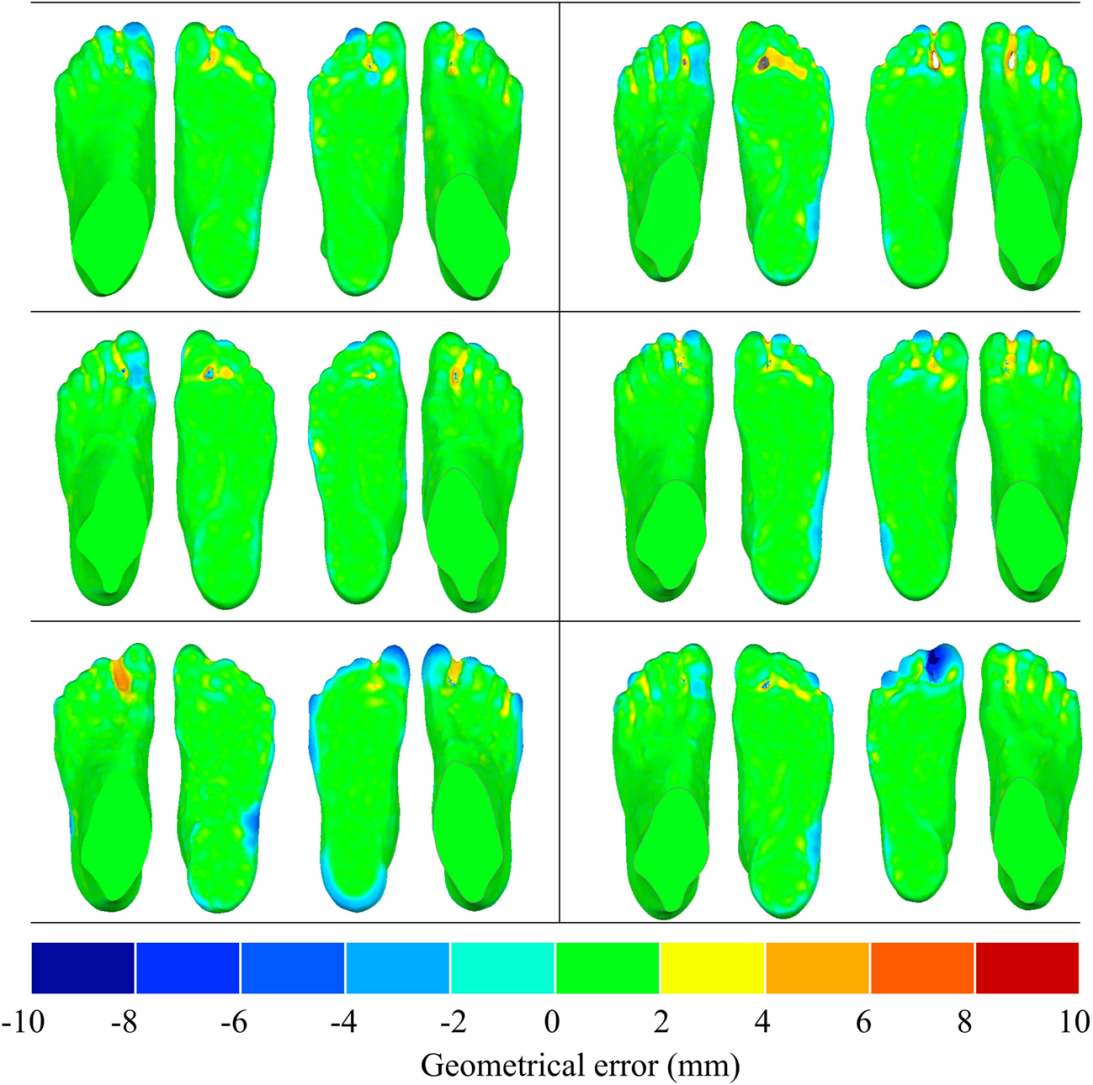
Geometric error maps between scaled and targeted foot surfaces for twelve feet

The geometric errors between the scaled and scanned foot surfaces were compared for the twelve feet (Fig. 8). The RBFPT and RBFST methods were implemented using the THI basis function, with 1000 sampling points employed for the RBFST method. The maximum error mainly occurred in the plantar side of the toes areas. For the maximum distance, the positive errors for the twelve feet were 7.1 ± 2.3 mm, and negative errors were -9.6 ± 2.8 mm. Regarding the average distances, the positive errors for the twelve feet were 0.17 ± 0.05 mm, and negative errors were -0.23 ± 0.09 mm.

### Barefoot foot pressures

**Fig. 9:**
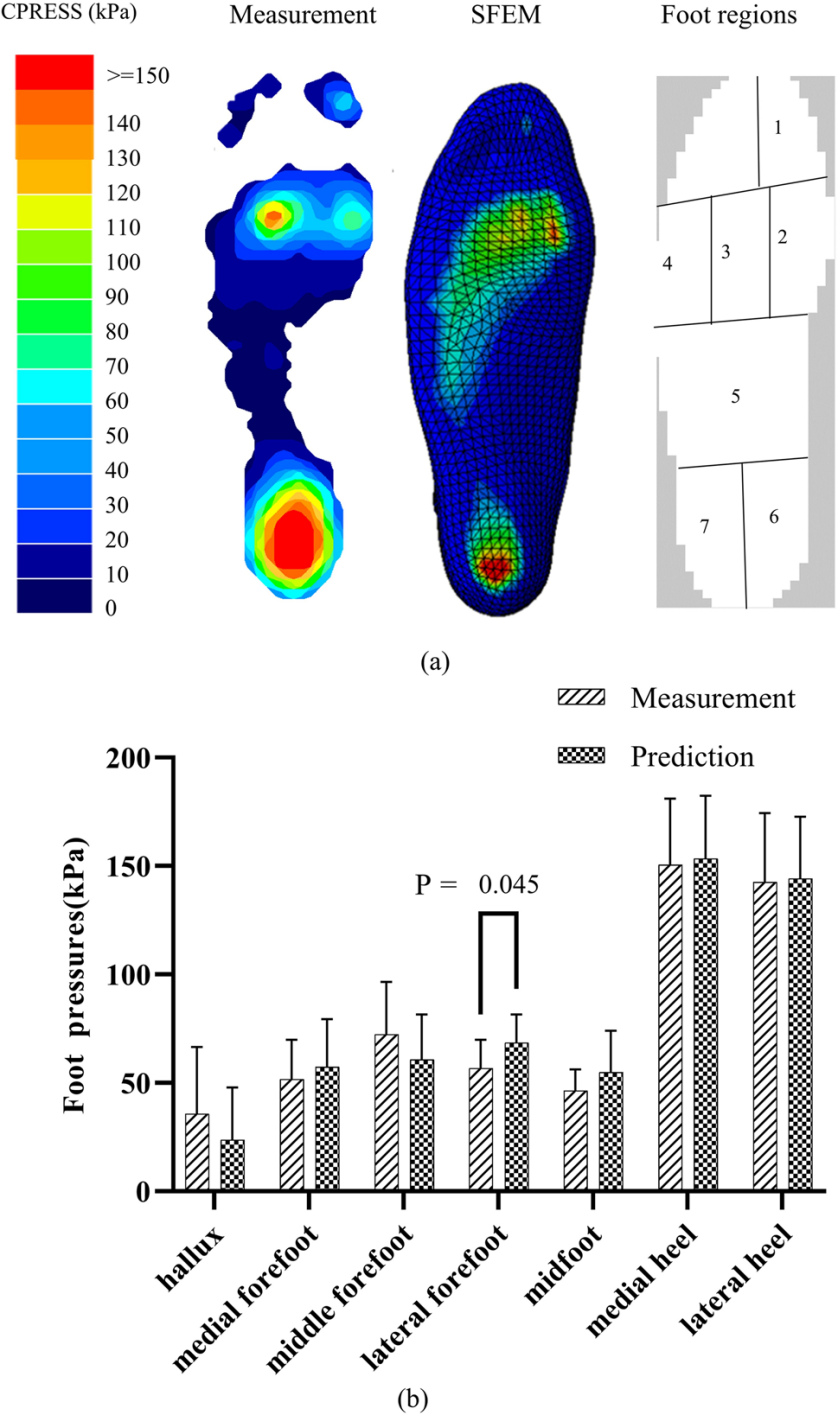
Validation of finite element model with the experimental measurement under barefoot conditions, including (**a**) foot pressure distribution and (**b**) statistically analysis. SFEM represents surface-based finite element model

The peak values of seven foot regions between the measured and predicted foot pressure were compared (Fig. 9(a)). There was no significant difference for the hallux, medial forefoot, middle forefoot, midfoot, medial hindfoot, and lateral hindfoot. In the lateral forefoot region, the measured peak values were significantly lower compared to predicted values (*p* = 0.045) (Fig. 9(b)).

### Foot-orthoses interface pressure

**Fig. 10:**
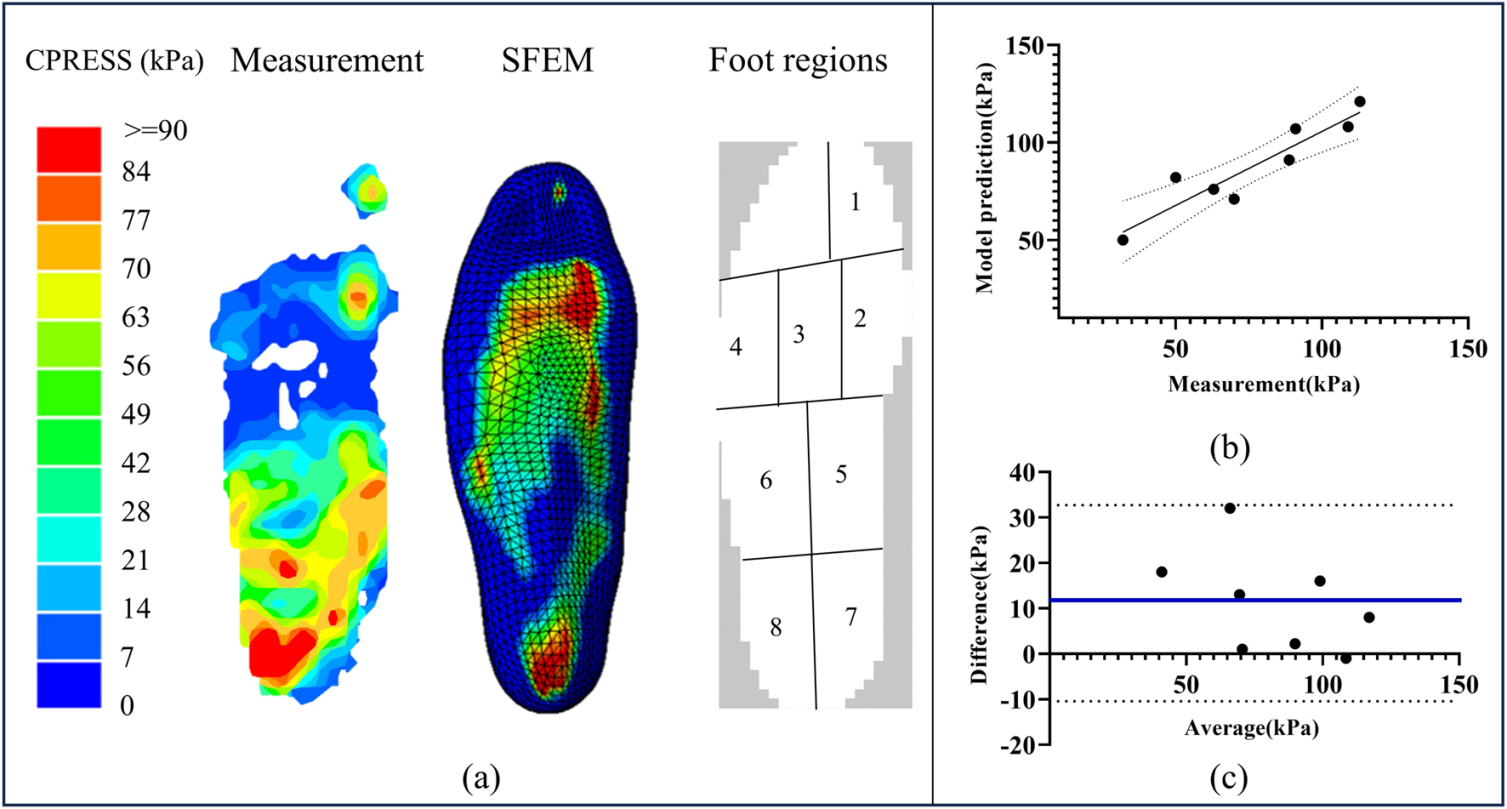
Comparison of measured and predicted foot-orthoses interface pressure, including (**a**) foot pressure distributions, (**b**) correlation analysis, and (**c**) Bland–Altman plot. The blue line represents the mean difference, and the dot lines are the 95% confidence interval

The foot-orthoses interface pressures among measurement and SFEM model prediction are compared in Fig. 10(a). The predicted and measured foot-orthoses interface pressures had a strong positive relationship (*R* = 0.92, *p* < 0.001). (Fig. 10(b)). The Bland–Altman plot showed a mean offset of 11.1 kPa (Fig. 10(c)). Based on the validation results, the proposed model prediction was considered to be reasonable.The peak pressures in the hindfoot were adopted in this study. The results showed that the SFEM has higher peak forefoot (107 kPa) and hindfoot pressure (121 kPa) when compared to the measured values (91 kPa in forefoot and 113 kPa in hindfoot). The SFEM model evaluated slightly higher foot-orthoses interface pressure values than measured, with a maximum deviation of 7.1%.

## Discussion

This study proposed the SFEM with the non-linear morphing algorithm, which could be used to predict the foot pressure distribution efficiently and accurately. Four customised scaling methods were adopted, namely 1) RBFPT, 2) RBFPT and RBFST with THI, 3) RBFPT and RBFST with MUL, and 4) RBFPT and RBFST with TRI. RBFPT and RBFST with the THI basis function achieved the best result. The averaged distances between the scaled and scanned foot surfaces were 0.23 ± 0.09 mm. Overall, there was no significant difference for the hallux, medial forefoot, middle forefoot, midfoot, medial hindfoot, and lateral hindfoot, except for the lateral forefoot region, the measured peak values were significantly lower compared to predicted values (*p* = 0.045). Meanwhile, the predicted and measured foot-orthoses interface pressures had a strong positive relationship (*R* = 0.92, *p* < 0.001).. The proposed SFEM method could be adopted to predict the foot-orthoses pressure with only scanned foot surfaces, which can provide guidance for pressure-relief foot orthoses design.

This study has adopted the non-linear morphing method to obtain the internal foot bony geometry that consisted of the SFEM along with the scanned foot surface. The non-linear morphing function was created according to the landmarks on the template and targeted foot surface [29]. The accuracy of the morphing function could be evaluated by the geometric error between the scaled and targeted foot surfaces. The results indicated that predicted foot surfaces overall agreed with the targeted scanned foot surfaces for the twelve feet. Based on the scaled foot bone and scanned foot surface, the SFEM can be more efficient and inexpensive than the MRI or CT-based foot modelling approaches. However, the local accuracy of foot geometry could be attenuated compared to realistic geometries. In this study, the geometric error in the forefoot part, especially for the plantar side of toes areas, was relatively large. It was in line with one previous study, which also adopted surface-based scaling methods to obtain the foot bony geometries and achieved relatively large errors in the phalange parts [37]. These errors could attribute to the position deviations in the toes during the scaling process or the lack of enough landmarks in the forefoot regions. The landmarks on the templated and targeted surfaces should be carefully selected to avoid manually induced landmarks errors [29]. Nevertheless, the proposed non-linear approach could construct foot surfaces with average distances of 0.23 ± 0.09 mm when compared to the scanned surfaces.

Various scaling methods were constructed by matching the landmarks between the different templates and targeted foot surfaces. The proper scaling functions could improve the construction accuracy of the scaled foot surface and foot bones. In this study, the RBFPT and RBFST with the THI basis function achieved the best result, which has also been adopted in the previous study [29]. Meanwhile, the number of sampling points could also affect the geometric errors in the RBFST process. It was considered that the geometric error between the scaled and targeted foot surfaces overall decreased with the increase of the sampling point numbers. Although the overall accuracy of the model could be improved, local errors especially in toe regions existed with the number of sampling points. The geometric error is the lowest at a sampling number of 1000 when the sampling points increase from 100 to 1500. The larger number of sampling points could increase the abrupt change of local regions, affecting the functional outcomes of scaled foot bony geometry [38]. Thus, the number of sampling points should be further evaluated for the RBFST function.

The pressure distribution between barefoot and ground was predicted based on the SFEMs among twelve feet. The peak foot pressures occurred in the hindfoot regions for the twelve feet, which were in line with previous studies [5, 39]. In this study, the internal foot bones were merged into one part, accelerating the calculation process compared to the detailed foot models [40]. There was no significant difference of peak values between predicted and measured six foot regions, except for the lateral forefoot region. The difference could be attributed to the geometry error resulting from the scaling procedure. It should be noted that the model with merged foot bones ignored the interaction between foot bones, muscles, ligaments, and cartilage, which could not be used to evaluate the internal foot biomechanical responses to various loading conditions. It should be carefully adopted for participants with severe foot deformity, such as rigid flatfoot and hallux valgus [41].

**Fig. 11:**
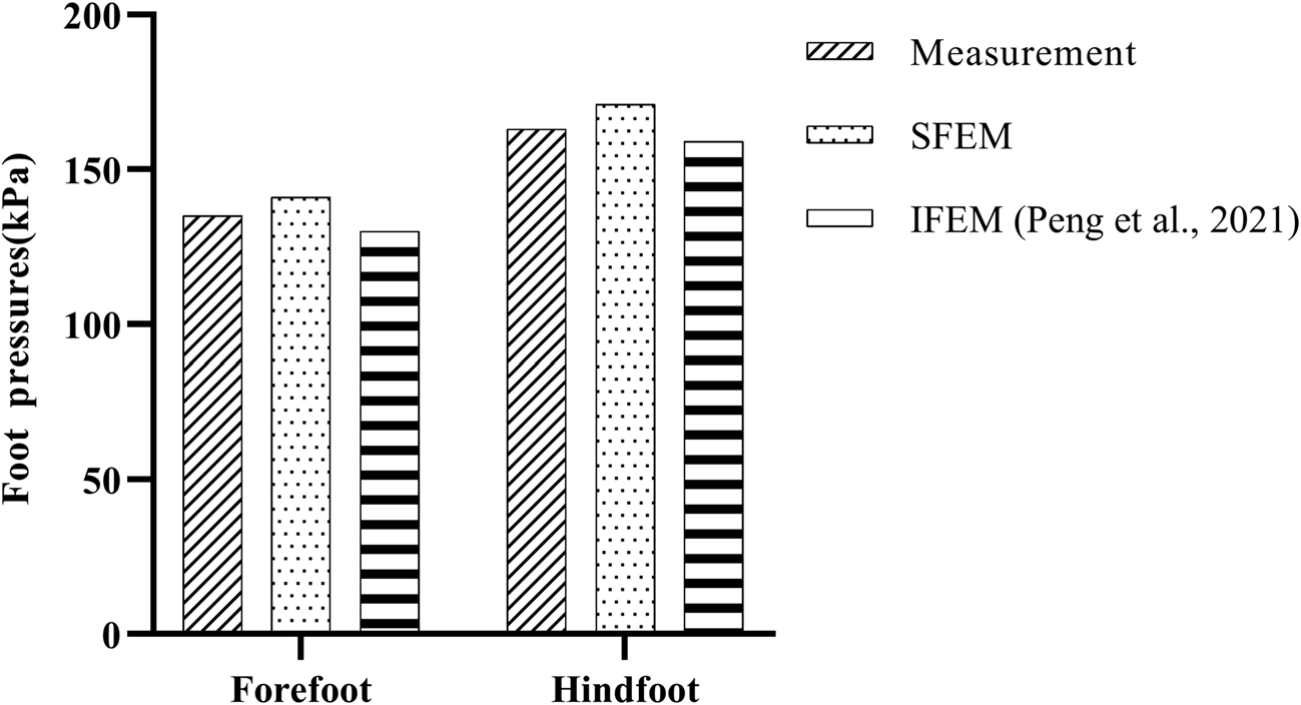
Comparison of foot pressure distributions among measurement, SFEM, and IFEM. SFEM represents the surface-based finite element model, IFEM represents the image-based finite element model. (source of IFEM: [26], under Creative Commons Attribution License)

The SFEM method were further evaluated and compared with measurements and predictions of image-based finite element model (IFEM) of our previous study [26] (Fig. 11). The SFEM adopted the same foot surface geometry boundary condtions, and material properties, except scaled bony geometries. The peak hindfoot and forefoot pressures were compared among the measurement, IFEM, and SFEM. The SFEM has the higher peak forefoot (141 kPa) and hindfoot pressure (171 kPa) than those of measurement (135 kPa in forefoot and 163 kPa in hindfoot) and IFEM (130 kPa in forefoot and 159 kPa in hindfoot).The SFEM merged the foot bones, which could not simulate the process of navicular drop. Therefore, the exterior loading exerted on the foot mainly acted on the forefoot and hindfoot region, accounting for the increased forefoot and hindfoot pressures. Meanwhile, the peak foot-orthosis pressure of SFEM were favorable compared to measurement, although the merged bony structure was adopted. One previous study also adopted the foot finite element model with merged foot bones and obtained reliable foot-orthoses interface pressure [23]. The proposed SFEM indicated the potential application in the pre-design of foot orthoses, which could partially anticipate the biomechanical effects of foot orthoses design.

In this study, several limitations should be noted. Firstly, the material properties used for the bony structures and soft tissues were obtained from previous literature, which could underestimate the prediction accuracy of the model. Evaluation of the customized foot soft tissue properties (stiffness and thickness) could be performed to improve the accuracy of the foot pressure prediction [42]. Secondly, this study has adopted the Glasgow-Maastricht foot model as the template when establishing the scaling function, which could ignore the population variation. Further studies should consider additional factors such as foot type, soft tissue thickness, and foot soft tissue properties to enhance the accuracy of foot pressure prediction. Moreover, it would be necessary to utilize different representative foot models as reference models when applying the method to participants with diverse foot structures and functional properties. Thirdly, the foot bones were merged into one segment, which could not simulate the translation of the internal foot bones. However, the predicted peak foot pressures overall agreed with measurements by using scanned foot surface-based modeling techniques. This study endeavoured to achieve a balance between accuracy and efficiency for customised foot orthosis optimisation [23, 25]. Fourthly, the proposed SFEM was only validated under the balance standing conditions, validation and evaluation on foot pressure under various activities will be performed in future study. Fifthly, we used linear tetrahedron elements instead of hexahedral elements to generate meshes for the bony and soft tissue structures. While linear tetrahedrons can be used to approximate the geometries, they may be less accurate and efficient than their hexahedral counterparts, especially in dynamic simulations [43]. Further study could consider the hexahedral elements in customized foot finite element modelling procedure.

## Conclusion

In this study, a fast and validated SFEM method was proposed based on the scanned foot surface geometry. The maximum geometric errors between scaled and targeted foot surfaces were relatively small except for the toes region. Meanwhile, the SFEM model could predict the foot pressure distributions for the twelve feet. The proposed SFEM model predicted slightly higher foot-orthoses interface pressure values than measured, with a maximum deviation of 7.1%. The proposed model could contribute to the biomechanical understanding of orthotic assessments, which could be applied in both clinical and commercial setting for treating foot pains.
